# Managing skin-related adverse events during mirdametinib treatment for NF1-PNs: expert guidance from the ReNeu trial

**DOI:** 10.3389/fonc.2026.1826735

**Published:** 2026-09-18

**Authors:** Angela C. Hirbe, Milan J. Anadkat, Christina L. Boull, Christopher L. Moertel, Molly Nickerson, Michael D. Weber, Dusica Babovic-Vuksanovic, Timothy R. Gershon

**Affiliations:** 1 Washington University School of Medicine, St. Louis, MO, United States; 2 University of Minnesota, Minneapolis, MN, United States; 3 SpringWorks Therapeutics, Inc., a Healthcare Company of Merck KGaA, Darmstadt, Germany; 4 Mayo Clinic Rochester, Rochester, MN, United States; 5 Emory University, Atlanta, GA, United States

**Keywords:** drug eruptions, mirdametinib, mitogen-activated protein kinase kinase inhibitors, plexiform neurofibromas, professional practice

## Abstract

**Background:**

Neurofibromatosis type 1 (NF1) is a genetic disorder in which plexiform neurofibromas (PNs) develop in approximately 30% to 50% of affected individuals, often causing substantial morbidity. Treatment of NF1-associated PNs is challenging due to disease variability and tumor complexity.

**Observations:**

In the Phase 2b ReNeu trial (NCT03962543), mirdametinib, a mitogen-activated protein kinase kinase 1/2 (MEK) inhibitor, demonstrated efficacy and a manageable safety profile in children and adults with symptomatic PNs, achieving confirmed objective responses in 52% of children and 41% of adults, with substantial reductions in target tumor PN volume and improvements in patient- and parent proxy-reported outcomes. However, dermatologic adverse events (AEs) are common with MEK inhibitors, including mirdametinib, and can impact adherence and quality of life. While the ReNeu protocol included supportive care considerations for skin reactions associated with mirdametinib, some investigators recognized a need for more detailed guidance. The ReNeu Scientific Steering Committee, in collaboration with oncology and oncodermatology experts, developed expert-driven recommendations for the proactive management of mirdametinib-related skin AEs.

**Conclusions:**

These strategies emphasize early recognition, individualized care, and timely intervention to help reduce the severity of mirdametinib-related skin AEs, minimize dose modifications, and support treatment continuity and long-term treatment goals. Although these recommendations were specifically developed for mirdametinib based on ReNeu trial experience, they may also provide practical guidance for managing skin AEs associated with other MEK inhibitors in clinical practice.

## 1 Introduction

Neurofibromatosis type 1 (NF1) is an autosomal dominant genetic disorder that occurs in approximately 1 in 2500 live births globally (1–4). Plexiform neurofibromas (PNs), a hallmark feature of the disease, are nonmalignant tumors that are reported in 30% to 50% of NF1 patients (2, 3, 5, 6). These tumors contribute significantly to the disease’s clinical manifestations, causing a range of morbidities, including pain, neurological deficits, physical dysfunction, and organ impairment (1). PNs are composed of multiple cell types, including Schwann cells, blood vessels, fibroblasts, macrophages, and mast cells; they can arise along any group of peripheral nerves and are often invasive (1). In NF1, the loss of neurofibromin, a tumor suppressor protein, results in dysregulated RAS activity and sustained activation of the mitogen-activated protein kinase (MAPK) pathway, which contributes to the abnormal growth and differentiation of cells associated with PNs (7).

Clinical trials have demonstrated the efficacy of mitogen-activated protein kinase kinase 1 and 2 (MEK) inhibitors in treating PNs, with two agents, selumetinib and mirdametinib, now approved by the US Food and Drug Administration (FDA), offering meaningful therapeutic options for patients with NF1-PNs (8–17). The pivotal, Phase 2b ReNeu trial (NCT03962543) established the efficacy and safety of mirdametinib, the first FDA-approved MEK inhibitor for the treatment of NF1 and symptomatic PNs not amenable to complete resection in both adults and children aged ≥2 years (8, 18–20).

In the ReNeu trial, mirdametinib was administered at a dose of 2 mg/m² twice daily (maximum dose of 4 mg twice daily) on an intermittent dosing schedule (3 weeks on/1 week off) (17). The primary endpoint was confirmed objective response rate, defined as the proportion of patients with a ≥20% reduction in the target PN volume on magnetic resonance imaging from baseline to cycle 24 (each cycle equals 28 days) on ≥2 consecutive scans within 2 to 6 months, assessed by blinded independent central review.

Among the 58 adults (≥18 years of age) and 56 children (2 to 17 years of age) enrolled, 41% of adults and 52% of children achieved a confirmed objective response, with a median best target PN volumetric change of -41% (range, -90% to 13%) in adults and -42% (range, -91% to 48%) in children. Notably, deep responses (>50% reduction from baseline in best target PN volume) were observed in 62% (15/24) of adults and 52% (15/29) of children with a confirmed response. Additionally, both cohorts reported significant and clinically meaningful improvements in patient- or parent proxy-reported outcomes, including worst tumor pain severity, pain interference, and health-related quality of life, which began as early as cycle 3 and were sustained throughout treatment.

Mirdametinib was generally well tolerated, with most adverse events (AEs) being grade 1/2 (mild-to-moderate). Dermatologic AEs associated with MEK inhibitors, including mirdametinib, are among the most commonly reported AEs in the clinical setting (9, 13–15, 17, 21–30). These AEs, even when low grade, can be dose-limiting, impacting patient adherence to treatment, health-related quality of life, and overall treatment outcomes (11, 13, 21, 24, 28, 30–34).

Although basic dermatologic supportive care guidance was provided in the multi-center ReNeu protocol, more detailed guidance was proposed for patients experiencing skin AEs during the course of the ReNeu trial. Following consultations with oncology and oncodermatology experts with extensive MEK inhibitor experience, detailed considerations for managing the most common skin adverse reactions were provided to sites and subsequently updated for inclusion in this manuscript.

The purpose of this manuscript is to present expert-driven strategies, developed by the ReNeu Scientific Steering Committee, which included ReNeu investigators and collaborating oncologists and oncodermatologists, for the prevention and management of mirdametinib-related skin reactions. These suggestions aim to provide healthcare professionals with practical strategies to help reduce the incidence and severity of skin reactions associated with mirdametinib while minimizing dose modifications, with the goal of ultimately supporting optimal treatment outcomes and continuity of care. Some of these recommendations could benefit all patients receiving MEK inhibitors.

## 2 Methods

The ReNeu Scientific Steering Committee collaborated with expert oncodermatologists and the principal investigator from a high-enrolling ReNeu trial site (n=12; ages 19 to 68 years) where no mirdametinib dose modifications were required due to skin treatment-related AEs (TRAEs), to develop guidance for managing these reactions. This guidance was developed following a thorough review of the literature and incorporation of expert dermatological knowledge. To inform the development of these expert recommendations, we conducted a targeted narrative literature review focused on MEK inhibitor–associated dermatologic adverse events and on the treatment of NF1-associated plexiform neurofibromas (PNs). We searched PubMed/MEDLINE using combinations of the following terms and their synonyms: “neurofibromatosis type 1,” “NF1,” “plexiform neurofibroma,” “MEK inhibitor,” “mitogen-activated protein kinase kinase inhibitor,” “mirdametinib,” “selumetinib,” “trametinib,” “binimetinib,” “dermatologic adverse event,” “cutaneous adverse event,” “skin toxicity,” “rash,” “dermatitis acneiform,” “acneiform rash,” “papulopustular eruption,” and “drug eruption.” Searches were limited to human studies and English-language publications and covered the period from database inception to June 2026 to capture key new data relevant to MEK inhibitors in NF1-PN. Clinical trials, larger observational series, systematic reviews, and clinical practice guidelines describing the efficacy, safety, and cutaneous toxicity profiles of MEK inhibitors, particularly in NF1-PN were prioritized. Reference lists of key NF1-PN and MEK inhibitor studies, related reviews, and major guidelines were also screened to identify additional relevant publications. Because the objective of this work was to develop pragmatic, expert-driven recommendations grounded in the ReNeu experience rather than to conduct a formal systematic review or meta-analysis, a protocol was not registered nor was PRISMA methodology applied. Instead, the literature was synthesized qualitatively and integrated with ReNeu trial data and expert clinical experience to inform the proactive management strategies presented in this manuscript.

In developing these recommendations, formal consensus methods (e.g., Delphi, modified Delphi, nominal group technique) were not used; instead, differing viewpoints were discussed during Steering Committee meetings and resolved by focusing on approaches that were consistent with the ReNeu protocol, supported by available evidence, and considered feasible in routine clinical practice. Draft recommendations and manuscript text were developed by a core writing group and reviewed by all authors; differences in interpretation were discussed and resolved by consensus. In cases where substantial uncertainty remained, recommendations were framed conservatively.

The ReNeu trial protocol included dermatologic supportive care suggestions and allowed for the use of medications to manage skin adverse reactions. Physical skin examinations were conducted regularly throughout the trial by study investigators and skin TRAEs were graded according to the National Cancer Institute Common Terminology Criteria for Adverse Events (CTCAE), version 5.0 (35). Routine dermatology consultation or confirmation was not mandated by the study protocol. While CTCAE terminology was used in this guidance, other terms may be used in routine oncology or dermatology practice. In this manuscript, CTCAE v5.0 terminology was used for standardized adverse event grading, whereas morphology-based clinical descriptors were used to further characterize cutaneous findings when detailed clinical information was available.

Per the ReNeu protocol, mirdametinib dosing was interrupted for grade ≥3 acneiform rash, with treatment resumed at the next lower dose if the reaction resolved to grade ≤1 or baseline within 14 days. For other AEs, dosing was interrupted for grade 2 events that were subjectively intolerable (excluding alopecia) and not controlled by supportive care, with treatment resumed at the same dose following resolution to grade ≤1 or baseline within 14 days. Similar dose modification guidance was later incorporated into the US prescribing information (36). Because of the low incidence of grade ≥3 paronychia observed in the ReNeu trial, the ReNeu Steering Committee did not create additional guidance specifically for its management.

### 2.1 Mechanisms of dermatological reactions

The MAPK signaling pathway plays a central role in regulating keratinocyte physiology, which is essential for normal skin (37). This pathway controls cellular processes, such as proliferation, differentiation, and apoptosis, integrating various signals to regulate keratinocyte differentiation and maintain healthy skin barrier function and integrity (37). Understanding the mechanism of action of MEK inhibitors is essential for recognizing the cause of potential dermatologic reactions (**Figure 1**).

**Figure 1:**
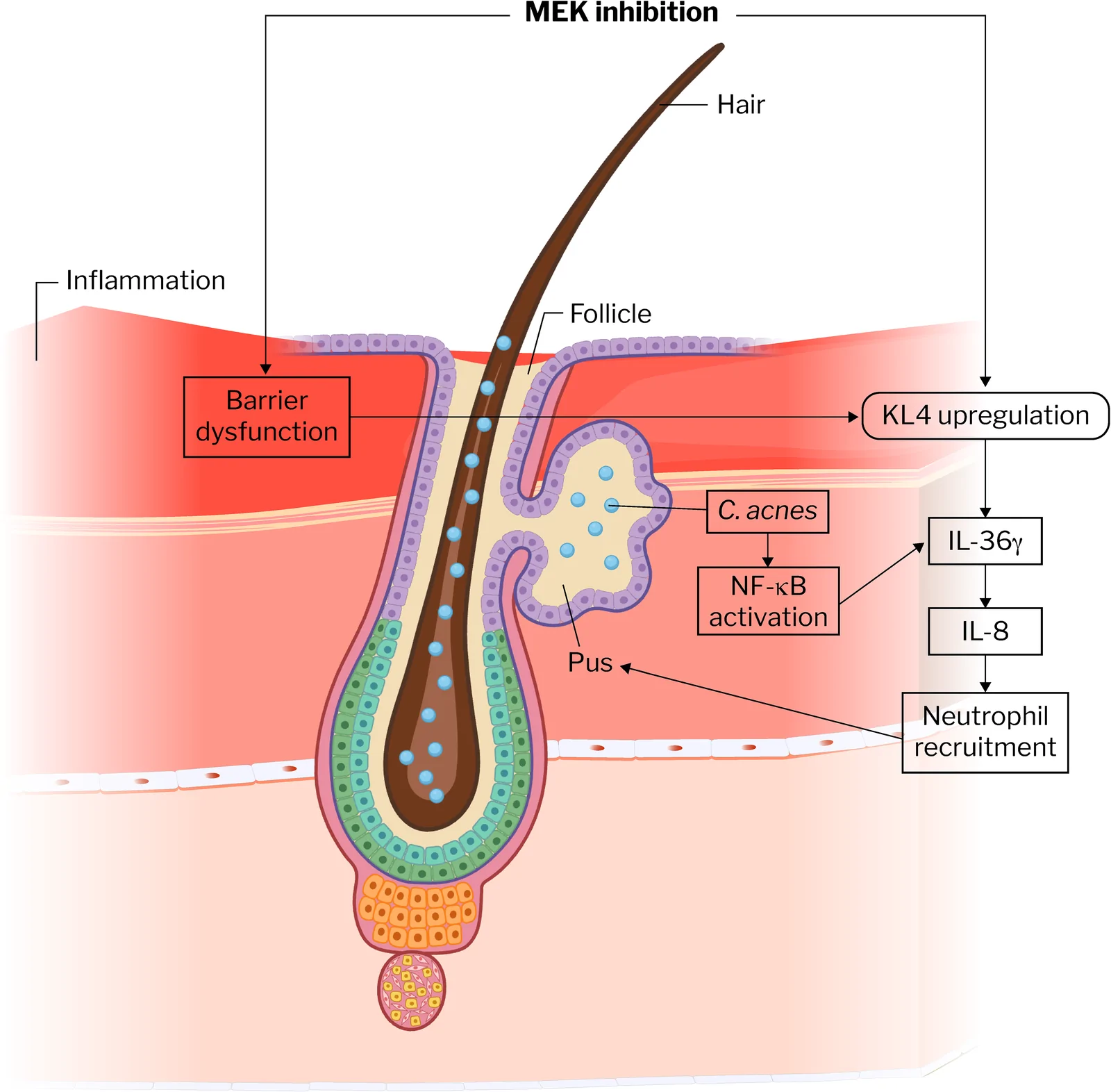
Mechanism of MEK inhibitor-induced skin reactions. MEK1/2 inhibitors can suppress MAPK pathway activity in nontumor tissues, including the skin, where they interact with the microbiome and impair the skin barrier and antibacterial defenses. MEK inhibition results in KL4 accumulation, which, together with NF-κB activated by *Cutibacterium acnes* through TLR2 signaling, binds the IL-36γ promoter to induce IL-36γ expression. This upregulation stimulates IL-8 production and neutrophil recruitment, manifesting clinically as a folliculocentric pustular eruption. *C. acnes, Cutibacterium acnes*; IL, interleukin; KL4, Krüppel-like factor 4; MEK, mitogen-activated protein kinase kinase; MAPK, mitogen-activated protein kinase; NF-kB, nuclear factor kappa-light-chain-enhancer of activated B cells; TLR2, toll-like receptor 2. Adapted from Satoh et al. (39) under a CC BY license.

MEK1/2 inhibitors, such as mirdametinib, suppress the overactive MAPK pathway within abnormal or tumor tissue. However, this targeted inhibition can also affect the MAPK pathway in nontumor tissues, most notably the skin, leading to unintended or “off-target” effects (38–40). In the skin, MAPK pathway suppression alters normal turnover and regeneration process, contributing to skin-related toxicities. In addition, MEK1/2 inhibitors interact with the skin microbiome, impairing the skin barrier and antibacterial defense mechanisms. This disruption of the skin microbiome, coupled with alterations in the MAPK pathway, triggers a neutrophil-rich inflammatory response through two mechanisms: (1) upregulation of the nuclear factor-κB transcription factor in keratinocytes, which leads to increased cytokine production (particularly interleukin [IL]-36γ and IL-8), and (2) the presence of *Cutibacterium acnes*, a bacterium prevalent in sebum-rich skin areas (**Figure 1**).

These changes in skin turnover and enhanced inflammatory response can result in clinical manifestations, including eczematous dermatitis, especially in prepubescent patients (**Supplementary Figures 1A–C**), and acneiform rash, particularly in postpubescent pediatric patients (**Supplementary Figures 1D, E**) (21–23, 38–42). An impaired skin barrier and compromised antibacterial defenses also contribute to the development of other dermatologic conditions, such as papulopustular rash, pruritus, xerosis, and folliculitis (**Figure 1**; **Supplementary Figure 1**) (40).

Similar to agents that target the epidermal growth factor receptor, skin reactions associated with MEK inhibitors typically appear on the face, scalp, and upper chest and back within the first few weeks of treatment (22, 39, 43–45). However, some patients may develop skin reactions later in treatment, and the exact timing can vary depending on the individual and the specific MEK inhibitor used (45–47).

### 2.2 Skin reactions associated with mirdametinib

In the ReNeu trial, most patients experienced at least 1 skin TRAE, with 93% of adults and 82% of children affected (**Table 1**; **Figure 2**). The majority of skin TRAEs reported in both adults and children were of grade 1–2 severity (**Table 1**; **Figure 2**). The frequency and types of skin TRAEs varied across different age groups (**Tables 1**, **2**; **Figures 2**, **3**). Dermatitis acneiform (also recognized clinically as acneiform dermatitis or acneiform eruptions) was the most commonly reported skin TRAE, with high frequencies observed in adults (93%) and postpubescent pediatric (88%) patients, while prepubescent (78%) patients had a notably lower incidence. Dry skin and rash were reported less frequently overall, with variation observed across the different age groups. Other skin events occurred at lower rates. Paronychia was more common in children (30%) than adults (2%), particularly in prepubescent children (34%; **Table 1**; **Figure 2**), which is consistent with the literature on MEK inhibitors (14, 24, 26, 48, 49). All cases were of grade 1–2 severity.

**Table 1:** Most common skin TRAEs in adults and children with NF1-PN in the ReNeu trial (17).

Patients, n (%)	Children (n=56)		Adults (n=58)	
	All grades	Grade 3	All grades	Grade 3
Total	46 (82)^a^	2 (4)	54 (93)	6 (10)
Dermatitis acneiform	24 (43)	1 (2)^b^	45 (78)	5 (9)^b^
Dry skin	8 (14)	0	8 (14)	0
Alopecia	7 (12)	0	7 (12)	0
Pruritus	3 (5)	0	5 (9)	0
Rash	7 (12)	1 (2)^b^	4 (7)	0
Eczema	6 (11)	0	2 (3)	0
Hair color changes	6 (11)	0	1 (2)	0
Paronychia	17 (30)	0	1 (2)	0

“Most Common” TRAEs includes ≥10% of patients in either cohort. There were no reported grade ≥4 skin TRAEs.

TRAE, treatment-related adverse event.

^a^ Some patients experienced more than 1 skin TRAE.

^b^ Grade 3 dermatitis acneiform and rash occurred in 1 postpubescent pediatric (ages 12 to <18 years of age) patient each, and in 5 young adults (ages 18 to ≤50 years of age).

**Figure 2:**
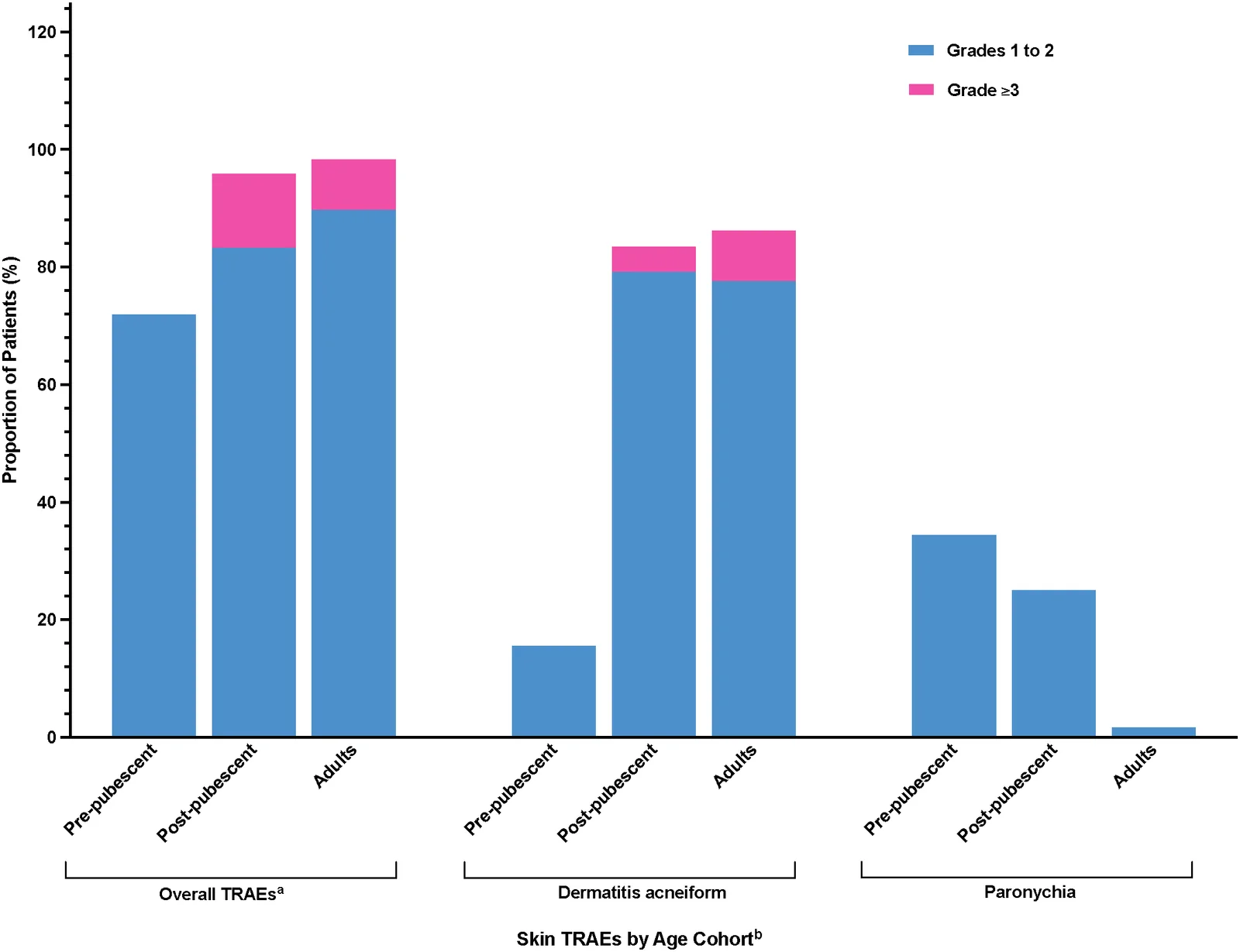
Proportion of most common (≥15% overall) skin TRAEs within each age group in the ReNeu trial (N = 114). Prepubescent, 2 to <12 years of age; n=32; postpubescent pediatric, 12 to <18 years of age; n=24; adults, >18 years of age; n=58. ^a^Some patients experienced more than 1 TRAE. ^b^Patients with at least 1 TRAE. TRAE, treatment-related adverse event.

**Table 2:** Most common skin TRAEs by age cohort in patients with NF1-PN in the ReNeu trial.

Patients, n (%)	Prepubescent patients (n=32)	Postpubescent pediatric patients (n=24)	Adults (n=58)
Skin TRAEs overall	25 (78)	21 (88)	54 (93)
Dermatitis acneiform	5 (16)	19 (79)	45 (78)
Dry skin	6 (19)	2 (8)	8 (14)
Rash	6 (19)	1 (4)	4 (7)
Patients with at least 1 skin TRAE leading to dose modification or discontinuation, n (%)			
Dose interruption	0	0	0
Dose reduction^a^	1 (3)	1 (4)	7 (12)
Permanent discontinuation^a^	2 (6)	2 (8)	8 (14)

“Most Common” TRAEs include ≥15% of patients in either cohort. Patients may have experienced more than 1 TRAE.

Age groups: prepubescent, 2 to <12 years of age; postpubescent pediatric, 12 to <18 years of age; adults, >18 years of age.

TRAE, treatment-related adverse event.

^a^ More than 1 skin TRAE could have contributed to dose reduction or permanent discontinuation.

**Figure 3:**
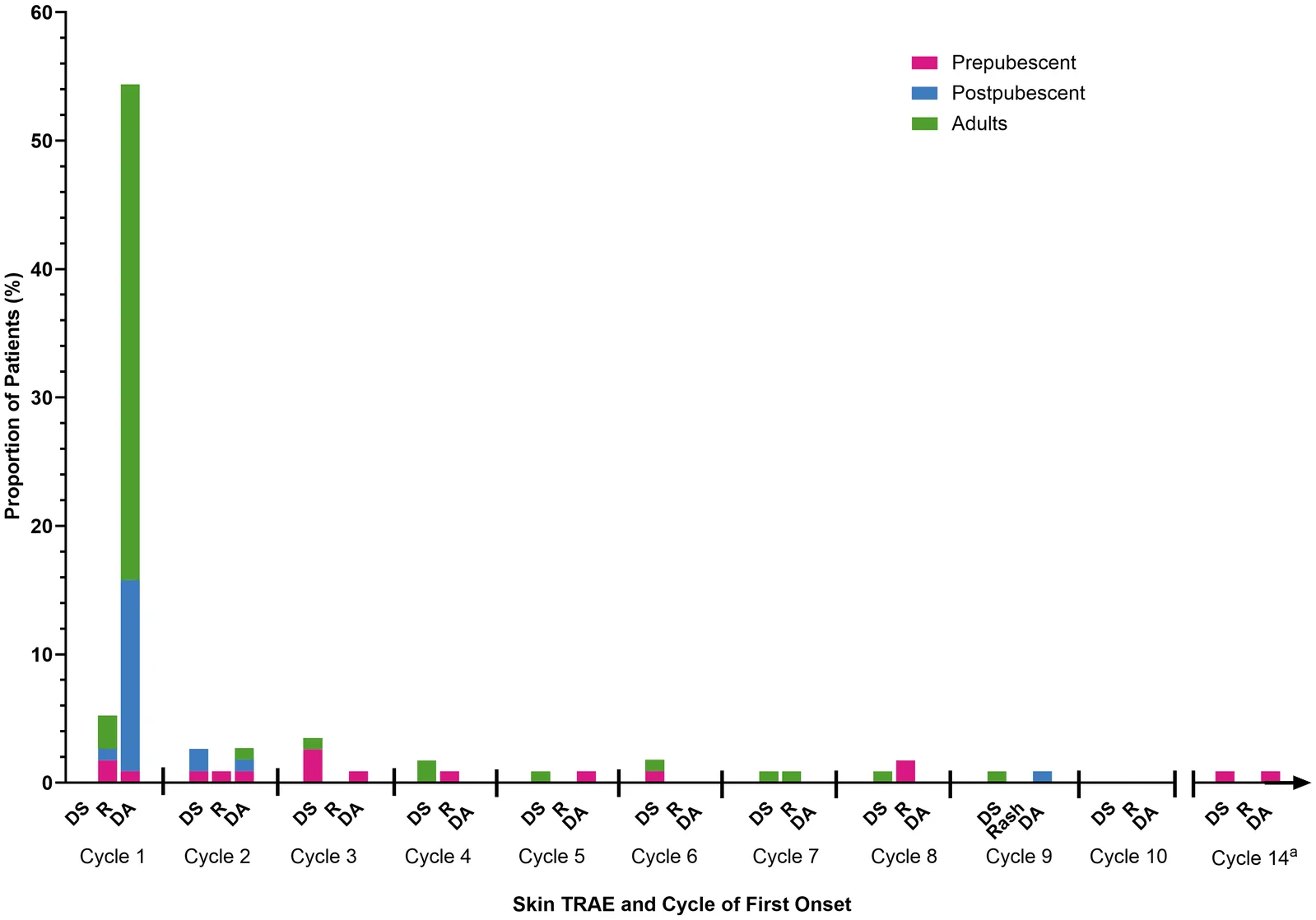
Cycle of onset of skin TRAEs in the ReNeu trial (N = 114). Prepubescent cohort, 2 to <12 years of age (n=32); postpubescent pediatric cohort, 12 to <18 years of age (n=24); adult cohort, >18 years of age (n=58). ^a^Patients were treated through cycle 49, however, no patients reported first onset of these most common skin TRAEs after cycle 14. DA, dermatitis acneiform; DS, dry skin; R, rash; TRAE, treatment-related adverse event.

Of the 114 adults and children, 71% (n=81) experienced a skin TRAE with first onset during cycle 1 or 2; the most common skin TRAEs with cycle of first onset is displayed in **Figure 3**. Among the 114 patients, 7% (n=8) experienced a skin TRAE of grade 3 severity, with no grade 4 or grade 5 skin TRAEs reported. These grade 3 cases were identified as dermatitis acneiform (9% [n=5] of adults and 2% [n=1] of children), rash (0% of adults, 2% [n=1] of children), and erythematous rash (2% [n=1] of adults, 0% of children).

Of the 114 patients, dose reductions due to skin TRAEs were observed in 7 (6%) adults and 2 (2%) children (**Table 2**). Permanent discontinuation due to dermatologic TRAEs occurred across all age groups: 6% (2/32) in prepubescent, 8% (2/24) in postpubescent pediatric, and 14% (8/58) in adult patients (**Table 2**). All pediatric discontinuations were due to grade 1–2 events, as were 6 of 8 (75%) adult discontinuations. Of the 4 pediatric discontinuations attributed to dermatologic TRAEs, 50% (n=2) occurred during Cycle 1, while 75% (n=6) of the 8 adult discontinuations occurred during Cycles 1 or 2. The median time to resolution for overall skin TRAEs was 47 days (range, 8 to 813 days).

Additional guidance on the management of paronychia was not proposed by the expert committee, although supportive care recommendations were included in the ReNeu trial protocol. These included warm soaks for acute, nonsurgical cases; oral antibiotics targeting *Staphylococcus aureus* when indicated; and incision and drainage for abscesses, with or without debridement, followed by targeted antibiotic therapy based on culture and sensitivity results.

### 2.3 Guidance for additional proactive management of mirdametinib-associated skin reactions

Skin-related AEs are the most frequent reactions observed in patients treated with MEK inhibitors, across both pediatric and adult populations (21). These reactions occur irrespective of whether the patient has NF1. The frequency of these dermatologic adverse events varies across different MEK inhibitors and patient populations, as reported in clinical trials. Unaddressed skin reactions associated with targeted agents can significantly affect a patient’s physical comfort, interfere with daily activities and work, impact emotional well-being, and lead to reduced treatment adherence, which may influence therapeutic outcomes (34, 50). Therefore, early identification and management of skin reactions are critical for optimizing patient care and treatment success.

#### 2.3.1 Risk assessment and preventive strategies

Before initiating mirdametinib treatment, it is advisable that healthcare providers perform a baseline skin assessment, including a detailed history of any pre-existing skin conditions, such as eczema, ichthyosis, or chronic dry skin, that may predispose patients to more severe skin reactions during treatment (C. Boull, personal communication) (30, 51). For these higher-risk patients, preventive measures, such as emollient-based skincare regimens and regular dermatologic monitoring, may be indicated. By addressing these risk factors proactively, healthcare providers can reduce the likelihood of additional dermatologic issues caused by the medication and optimize the patient’s ability to tolerate mirdametinib treatment.

#### 2.3.2 Proactive skincare for patients of all ages

For patients receiving mirdametinib, a comprehensive approach that includes patient education, proactive skincare, regular monitoring, and early intervention is recommended for mitigating MEK inhibitor-associated TRAEs (21). On the basis of experiences from the ReNeu trial, patients are advised to optimize general skincare to prevent and limit dermatologic reactions including dry skin. Daily baths using mild, hypoallergenic cleansers may be helpful. Soap should be limited to essential areas (eg, face, axillae, groin) to reduce the risk of skin irritation. Dry skin, if left untreated, can increase the risk of injuries and fissures, which can lead to infections, making regular moisturizing essential for maintaining skin integrity (21, 52). Therefore, application of emollient-based creams or ointments at least twice daily helps maintain skin hydration and prevent dryness. Avoidance of topical retinoids and other skin irritants, such as benzoyl peroxide, salicylic acid, acne washes, scrubs, exfoliants, anti-aging creams, and alcohol-based products, may be considered, as these agents can exacerbate skin dryness.

#### 2.3.3 Age-specific considerations

Skin reactions associated with mirdametinib may present differently in prepubescent and postpubescent pediatric patients, necessitating tailored management strategies (**Figure 4**) (41, 42, 53).

**Figure 4:**
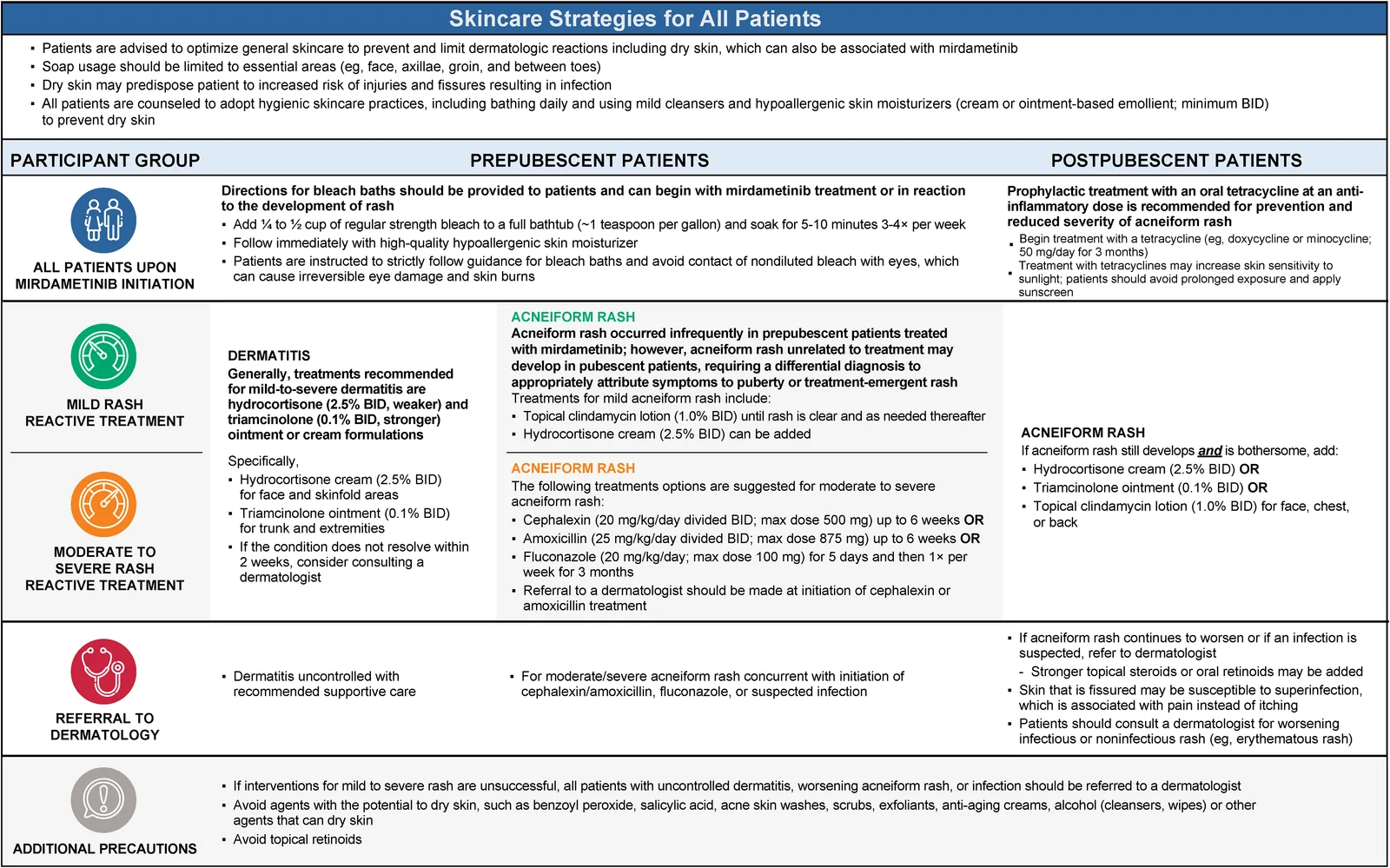
Skincare management strategies for ReNeu trial patients. Adapted from Hirbe A, et al. (42), with permission.

##### 2.3.3.1 Prepubescent patients

In prepubescent patients, the primary dermatologic reactions are often eczematous in nature (33, 41). For these patients, proactive management can include the use of diluted bleach baths, particularly when initiating mirdametinib or when a rash develops. It has been demonstrated that bleach baths have antimicrobial and antibiofilm properties, as well as anti-inflammatory and antipruritic effects (54). The recommended bleach concentration is one-fourth to one-half cup of regular-strength bleach in a full bathtub of water (approximately 1 teaspoon of bleach per gallon of water). Patients should soak for 5–10 minutes, 3–4 times a week (21). However, patients and caregivers should be cautioned to avoid direct contact with undiluted bleach, as it can cause irreversible eye damage and skin burns (55). Splashless and scented bleach formulations should be avoided.

Although acne vulgaris in prepubescent patients is less common, it can still occur, while acneiform dermatitis is typically medication-induced. Therefore, any acne-like eruptions in these patients should be carefully evaluated to distinguish between baseline acne and treatment-related dermatologic toxicity, as treatment with a MEK inhibitor could potentially exacerbate the condition (56).

Treatments for dermatitis depend on severity. For mild or moderate-to-severe dermatitis, topical treatments are commonly used in practice. Hydrocortisone cream (2.5%) may be considered for the face and skinfold areas, while triamcinolone ointment (0.1%) may be used for the trunk and extremities. For mild dermatitis acneiform, topical clindamycin lotion (1.0%) may be applied twice daily; hydrocortisone cream (2.5%) twice daily may be considered if needed. For moderate-to-severe dermatitis acneiform, the following oral agents are proposed:

- Cephalexin, 20 mg/kg/day divided BID (max 500 mg, ≤6 weeks),
- Amoxicillin, 25 mg/kg/day divided BID (max 875 mg, ≤6 weeks),
- Oral fluconazole, 20 mg/kg/day (max 100 mg, 5 days then once weekly for 3 months) (57).

Although fluconazole is a strong cytochrome P450 isoform 3A4 inhibitor, it is noteworthy that mirdametinib is not primarily metabolized through the cytochrome P450 enzyme. As a result, no interaction between fluconazole and mirdametinib is anticipated, and dose modifications should not be needed (36). In cases where oral agents are prescribed for moderate to severe dermatitis acneiform, referral to a dermatologist is recommended to ensure optimal management. If conditions worsen despite treatment, further referral for specialist evaluation is advised to prevent complications and ensure effective intervention. For those inexperienced with MEK inhibitors, partnering with a dermatologist may be beneficial in managing more challenging cases.

##### 2.3.3.2 Postpubescent pediatric patients

Postpubescent pediatric patients treated with MEK inhibitors are more likely to develop acneiform rash, especially during the first 2 cycles of mirdametinib treatment, due to maturation of sebaceous glands and changes in skin flora associated with puberty (24, 33, 41, 53). To mitigate this risk, proactive management strategies including oral doxycycline or minocycline at an anti-inflammatory dose (50 mg/day) may be considered for 3 months, starting concurrently with mirdametinib, to reduce the severity of dermatitis acneiform (53, 58, 59). Oral doxycycline is an option in patients over the age of 8 years, but it is associated with a risk of impaired tooth development and permanent dental staining for younger patients (60). Because of the risk of photosensitivity, patients taking tetracyclines should minimize prolonged sun exposure and use protective measures such as clothing and sunscreen (60).

For patients with persistent dermatitis acneiform the following strategies are proposed: hydrocortisone cream to the face (2.5% BID); triamcinolone ointment (0.1% BID) to the trunk and extremities (excluding the face); or topical clindamycin lotion (1%) to affected areas such as the face, chest, or back. In selecting a topical steroid, potency is a key consideration. Hydrocortisone 2.5% is the least potent, while triamcinolone 0.1% is considered medium potency (61). The choice of vehicle (cream or ointment) may depend on patient preference; however, lotions or solutions are recommended for use on areas with hair.

Patients should consult a dermatologist if they experience worsening rashes—whether infectious or noninfectious (eg, pustular rashes or erythematous)—or if dermatitis acneiform worsens or infection is suspected. Stronger topical steroids or oral retinoids may be added in such cases. Importantly, if the skin becomes fissured, superinfection with an accumulation of crusts and purulent material can occur, which is often associated with pain rather than itching (62).

## 3 Discussion

The expert guidance developed and refined by the ReNeu Scientific Steering Committee, in collaboration with oncologic and oncodermatologic experts, provides actionable steps to help minimize discomfort, support continued treatment, and potentially improve the overall treatment experience for patients with NF1-PN. These suggestions build upon existing guidance and standard clinical practice, providing a unified, proactive framework for managing dermatologic TRAEs. Our recommendations are also broadly consistent with recently published expert consensus guidance on MEK inhibitor–associated dermatologic toxicities, while extending these general principles to the NF1-PN setting by incorporating age-stratified ReNeu data (63–65). The spectrum of dermatologic TRAEs observed in ReNeu is generally consistent with the dermatologic toxicities reported with MEK inhibitors, including acneiform dermatitis, rash, xerosis, and paronychia (10). However, because available evidence is derived from separate clinical studies with differing patient populations, study designs, dosing schedules, supportive care practices, and adverse event reporting, direct comparisons among individual MEK inhibitors should be interpreted with caution.

Although these recommendations were developed in the context of the ReNeu trial evaluating mirdametinib, they may have broader applicability to patients receiving other MEK inhibitors. Caution is advised when co-administering supportive medications such as fluconazole, as these may interact with some MEK inhibitors that are primarily metabolized by cytochrome P450 isoform 3A4 (10).

The ReNeu trial demonstrated that mirdametinib offers significant and sustained benefits for both children and adults with NF1-PN, showing deep and durable PN volume reductions as well as improvements in patient- and parent proxy-reported outcomes over time. Notably, the median time to the best target PN volumetric change from baseline was 13.4 months (range, 4.0-32.7 months) in children and 15.2 months (range, 4.0-40.0 months) in adults. Additionally, 57% of children reported meaningful reductions in worst tumor pain severity, and 39% reported improvements in health-related quality of life (HRQOL) by cycle 5. These findings increased to a reduction of 73% for worst tumor pain severity and a 45% improvement in HRQOL by cycle 13, suggesting that therapeutic benefits may take time to fully manifest. Furthermore, high participation rates in the long-term follow-up phase among those who completed the treatment phase, 85% in children and 84% in adults, suggest that patients recognize the benefits of continued treatment despite potential adverse events.

A significant challenge during mirdametinib treatment, as seen in the ReNeu trial, is the onset of dermatologic TRAEs. These reactions frequently develop in the first 2 cycles, with 71% of patients experiencing the first onset of a skin TRAE during cycle 1 or 2 (**Figure 3**). Although most of these events were grade 1–2 in severity (**Table 2**), with only 7% of patients experiencing a grade 3 skin TRAE, these dermatologic events can still significantly impact patients. Setting clear expectations and engaging in early discussions about managing dermatologic TRAEs are critical for improving patient outcomes and treatment adherence.

An analysis of dermatologic TRAEs by age cohorts revealed that postpubescent pediatric (96%) and adult (78%) patients experienced the highest rates of dermatitis acneiform (**Table 1**), consistent with previous reports in the literature (24, 33, 47, 49, 66). Of note, younger adults (18 to ≤50 years of age) exhibited higher rates of dermatitis acneiform compared to older adults (≥50 years of age); however, given the overall consistency of dermatologic findings across the adult population, detailed subgroup analyses are not presented. Postpubescent pediatric and adult patients also accounted for all grade 3 dermatologic TRAEs. In contrast, prepubescent patients experienced a higher prevalence of any-grade rash (19%) compared with postpubescent pediatric (4%) and adult (7%) patients (**Table 2**). While most skin TRAEs were grade 1–2 in severity (**Table 1**; **Figure 2**), the majority (75%) of discontinuations due to dermatologic TRAEs among adults were attributed to grade 1–2 severity. Consistent with this observation, treatment discontinuation due to dermatologic TRAEs occurred more frequently in adults (14%) than in prepubescent (6%) or postpubescent (8%) pediatric patients (**Table 2**). Taken together, these findings suggest that adult and postpubescent pediatric populations may be more susceptible to the dermatologic toxicities associated with MEK inhibitors, potentially impacting treatment tolerability in these patients.

Acneiform rash and related folliculocentric eruptions likely represent on-target pharmacodynamic effects of MEK pathway inhibition in the skin; however, ReNeu was not designed to evaluate these events as biomarkers of systemic efficacy, and no conclusions can be drawn regarding their predictive value (64).

Time to resolution of dermatologic TRAEs was variable. The variation in resolution time may be attributable to the built-in week-off in mirdametinib’s intermittent dosing schedule, which could allow TRAEs to improve without requiring treatment modification. However, the ReNeu trial was not designed to evaluate the effect of the intermittent dosing schedule on dermatologic toxicities, and no conclusions regarding this potential relationship can be drawn from these data. This highlights the individualized nature of dermatologic TRAEs and suggests that proactive supportive care and an intermittent dosing schedule may contribute to patient tolerability. Healthcare providers can help manage these reactions effectively and potentially optimize the long-term benefits of treatment.

Proactive and ongoing management strategies, such as moisturizers and oral antibiotics, have shown benefit in mitigating dermatologic TRAEs associated with targeted agents in general (21, 53, 57–59). In a recent retrospective study, a subset of pediatric patients treated with trametinib (n=5) received doxycycline prophylaxis (100 mg once or twice daily for 3 months, starting at or just before MEK inhibitor initiation) (65). Those who received the prophylaxis still experienced acneiform eruptions but did not require MEK inhibitor modifications or discontinuation due to paronychia or acneiform eruptions. Interestingly, none of the patients receiving doxycycline prophylaxis developed paronychia.

Several limitations of the current guidance developed and refined by the ReNeu Scientific Steering Committee should be noted. Suggestions for managing dermatologic TRAEs, based on expert clinical experience, were introduced later in the ReNeu trial, after the original protocol was developed, and their adoption was not mandated across study sites. Although this modification reflects the adaptive nature of clinical trial design in response to emerging patient needs, the lack of inclusion in the initial protocol limited systematic evaluation of their impact on dermatologic TRAEs. Evidence of efficacy remains anecdotal due to the absence of specific outcome measures and prospective validation across several key aspects of dermatologic TRAE management, highlighting the need for further investigation into the long-term effects of these strategies. This includes the prophylactic use of systemic antibiotics for moderate-to-severe acneiform rash, which has not been prospectively studied. While there is historical precedent for the use of clindamycin lotion in the management of acneiform rashes (44), further research is needed to evaluate its efficacy in the setting of MEK inhibitor-associated acneiform rash.

There was potential for bias in patient-reported outcomes, as subjective assessments of skin-related side effects may not always align with objective clinical evaluations, potentially leading to over- or under-reporting of adverse events. Cutaneous reactions in ReNeu were primarily documented by oncologists and graded using CTCAE v5.0, and dermatology consultation was not mandated per protocol. As a result, the predominance of oncologist-reported events may have led to under-recognition or misclassification of specific morphologies (eg, differentiating eczematous dermatitis from early acneiform or folliculitis-like eruptions), and differences in severity grading between oncologists, dermatologists, and patients may further contribute to variability in reporting. The use of broad terms such as “rash” likely underestimates the true prevalence of distinct clinical entities (eg, papulopustular eruptions, xerotic dermatitis), limiting the precision with which our recommendations can be mapped to specific phenotypes. Future MEK inhibitor studies in NF1-PN should prospectively incorporate dermatology co-assessment, including standardized photo documentation and morphology-based classification, to improve the accuracy and reproducibility of skin AE characterization. Despite these limitations, the high frequency of skin TRAEs, their early onset (often within the first 2 cycles), and their impact on dose modification and discontinuation in ReNeu are robust findings, underscoring the importance of proactive, multidisciplinary management.

## 4 Conclusion

The ReNeu trial established the treatment benefit of mirdametinib for NF1-PN. Dermatologic events were the most common TRAEs, particularly dermatitis acneiform in postpubescent pediatric and adult patients. Most skin TRAEs were grade 1-2, emerged within the first two treatment cycles, and had variable times to resolution. Effective management, including the proactive measures described here, is essential for maintaining adherence and maximizing treatment benefits. Proactive strategies, early intervention, and individualized care are key to addressing skin reactions and enabling patients to continue treatment toward achieving their NF1-PN-related treatment goals. Given that skin TRAEs often precede therapeutic benefits, setting appropriate patient expectations, implementing proactive skincare, identifying at-risk patients early, and regular monitoring may mitigate the impact of dermatologic TRAEs on patient well-being and treatment adherence.

## Supplementary material

Supplementary Figure 1 Typical MEK Inhibitor-Induced Skin AEs From Pre- and Postpubescent Pediatric Patients (42). Patients shown here received MEK inhibitors outside the ReNeu study. **(A)** Papular variant of dermatitis on prepubescent patient; **(B)** plaque of dermatitis on the anterior neck and chest of prepubescent patient; **(C)** acneiform rash on young postpubescent pediatric patient; **(D)** grade 2 rash on adult patient; **(E)** rash on adult patient. Reprinted with permission from Milan J. Anadkat and Christina Boull.
