# Supplementary material for: Managing skin-related adverse events during mirdametinib treatment for NF1-PNs: expert guidance from the ReNeu trial
Source: Front Oncol. 2026 Sep 18;16:1826735. doi: 10.3389/fonc.2026.1826735 (PMC13631732; doi:10.3389/fonc.2026.1826735)
Supplement: Supplementary file 1 [file DataSheet1.docx]

# SUPPLEMENT

**SUPPLEMENTARY FIGURE 1**

Typical MEK Inhibitor-Induced Skin AEs From Pre- and Postpubescent Pediatric and Adult Patients.^1^

Patients shown here received MEK inhibitors outside the ReNeu study. **A**, Papular variant of dermatitis on prepubescent patient; **B**, plaque of dermatitis on the anterior neck and chest of prepubescent patient; **C**, acneiform rash on young postpubescent pediatric patient; **D**, grade 2 rash on adult patient; **E**, rash on adult patient. Reprinted with permission from Milan J. Anadkat and Christina Boull.

**Reference**

1. Hirbe A, Anadkat MJ, Boull C, et al: Addressing skin adverse events during mirdametinib treatment in patients with neurofibromatosis type 1-associated plexiform neurofibromas: Guidance from a multidisciplinary group of experts involved in the ReNeu trial. Presented at the 29th Annual Meeting of the Society for NeuroOncology, November 21-24, 2024, Houston, TX.
